# Development and psychometric analysis of a new tool to assess food literacy in diabetic patients

**DOI:** 10.1186/s40795-022-00626-4

**Published:** 2022-11-16

**Authors:** Fatemeh Bastami, Mahnaz Mardani, Pouria Rezapour

**Affiliations:** 1 Nutritional Health Research Center, School of Health, Lorestan University of Medical Sciences, Khorramabad, Iran; 2 Department of Nutrition, Nutritional Health Research Center, Lorestan University of Medical Sciences, Khorramabad, Iran; 3 Kharazmi University, Tehran, Iran

**Keywords:** Food literacy, Diabetes, Psychometrics, Content validity, Construct validity

## Abstract

**Background:**

One of the factors affecting self-care in diabetic patients is food literacy, which helps said patients in following a healthy diet. Thus, it is crucial to analyze food literacy in diabetic patients through suitable and reliable instruments.

**Objective:**

The current study aimed to design a questionnaire for food literacy assessment in diabetic patients and analyze its psychometric features.

**Method:**

The present study was a cross-sectional descriptive analysis carried out in 2021. Firstly, the concepts of food literacy in diabetic patients were identified and the questionnaire was deigned based on them. Secondly, its face and content validities and its reliability were analyzed. Finally, the construct validity was analyzed by exploratory factor analysis. The study was carried out on 300 diabetic participants chosen at random via stratified cluster sampling from Health service centers. The exploratory factor analysis was carried out by extracting the main factors and using varimax rotation with eigenvalue values more than 1.

**Results:**

A five-pronged structure accounted for 52.745% of food literacy variance. This included the ability to read food facts, practical ability to group foods, the ability to identify the caloric content of different foods, the ability to understand the effect of food on health, and the ability to prepare a healthy meal. Items with an impact score below 1.5 were discarded. Additionally, items with CVR scores below 0.62 and CVI scores below 0.79 were deleted too. The Kaiser-Meyer-Okin measurement was 0.836 (*p* < 0.001). Alpha Cronbach Scale dimension was 0.610–0.951.

**Conclusion:**

The results of this study showed that the exploratory dimensions of the current study were consistent with health literacy measurements, such as functional, interactive, and critical food literacy. This scale has acceptable reliability and validity. Health professionals can use this scale to analyze and improve food literacy in diabetic patients. This is a new instrument and thus far no questionnaire has been made to evaluate food literacy in diabetic patients.

**Supplementary Information:**

The online version contains supplementary material available at 10.1186/s40795-022-00626-4.

## Introduction

Food literacy is a combination of knowledge, skills, and needed behaviors for planning, managing, choosing, preparing, and consuming food in order to satisfy nutritional needs and to make correct nutritional choices. Improving food literacy is acknowledged as being a key component of choosing healthy foods and sustaining a healthy nutritional lifestyle and thus prevention of non-contagious diseases [1].

Food literacy is closely associated with the notion of health literacy [2]. A systematic review study showed that food literacy is recognized as a special form of health literacy [2]. Low levels of health literacy have a negative impact on providing care for and treating chronic diseases such as diabetes. Patients afflicted with a disease such as diabetes need specific lifelong self-care. Based on previous studies, health literacy was of key importance for the studied patients in following a specific diet [3, 4]. Based on some studies, diabetic patients with inadequate health literacy are less aware of their disease and most likely do not follow advice given by health professionals and do not follow written instructions regarding self-care. They do not usually take responsibility for their own health and thus control on their blood glucose levels poorly [5]. A previous study showed a meaningful statistical relationship between health literacy and following the dietary aspects of self-care [6]. Based on previous studies, dietary behaviors and dieting are of key importance in controlling blood glucose levels in diabetic patients. In order to properly control their blood glucose levels, diabetic patients should adopt proper dietary behaviors. To attain this, patients should be taught about food literacy [7, 8]. In Iran, Gabrik’s pyramid is used to educate diabetic patients [9, 10].

Studies have shown that despite extensive instruction, many diabetic patients do not pay enough attention to self-care and do not follow healthy dietary habits. Self-care is affected by multiple factors such as food literacy [4, 6].According to previous studies, awareness of the relationship between nutrition and blood glucose levels can lead to healthy dietary behaviors in diabetic patients. Some patients are aware of the relationship between their diet and improving blood glucose levels, and accordingly readily adopt the diet suitable for a diabetic patient. There is a significant relationship between a patient’s understanding of the benefits of a healthy diet and their adoption of the related behaviors [11]. Previous studies have shown that improved food literacy has a positive effect on dietary behaviors and well-being [3, 8].

Factors that affect a diabetic patient’s self-care behavior include food literacy and following a healthy diet [12]. Previous studies on food literacy have not focused on the effects of healthy diets, but have only taken general health literacy into account [3, 13–15]. Although there has been an increase in studies carried out in this field, there has been limited progress due to the lack of an accepted method for measuring food literacy. Thus, in order to guide the advancement of knowledge and ensure the effectiveness of nutritional interventions, a scale should be designed to analyze food literacy levels and the diets of diabetic patients [14]. The present study was carried out to develop a questionnaire to measure factors related to food literacy of diabetic patients and to analyze its psychometric features.

## Methods

The current methodological study aimed to develop and validate a questionnaire on food literacy of diabetic patients. The study setting was comprehensive health service centers in Khorramabad, Lorestan, Iran. The current methodological study essentially includes the following steps:

1. Assessing the extent of food literacy and its component parts (items of the scale).
2. Testing the questionnaire’s reliability and validity [16].

### Development of the scale

**Phase 1**: Assessing the concepts and components of food literacy (items of the scale).

A Literature review: This was done by a general search and review of previous studies carried out in different countries and societies. This was carried out by conducting searches in the PubMed, ISI, Science Direct, Scopus, and Google Scholar indices. This helped in identifying the concepts and components of food literacy. To carry this out the following keywords were used: food skills, food literacy, nutritional literacy, health literacy, food preparation, food choice, diabetes, and food welfare.
B The item pool regarding food and nutritional literacy of diabetic patients was established based on the guidelines and known elements and findings of previous studies and scientific texts [17–22]. The items incorporated in the questionnaire were compiled by a group of 10 experts and specialists from different fields, including health education (3 people), nutrition (5 people), nursing (1 person), statistics (1 person). Great care was taken to write the items clearly with precise phrasing. This method resulted in the formation of a questionnaire made up of 88 items used to analyze the parameters related to food literacy of diabetic patients

**Phase 2**: Testing the questionnaire’s validity  and reli­ability. All the stages of validity and reliability of the questionnaire were performed on people with type 2 diabetes referring themselves to comprehensive health service centers in Khorramabad city, Lorestan, Iran.

### Face validity

Both qualitative and quantitative methods were used to determine face validity. In the qualitative method, 20 people (including 10 women and 10 men) who were similar to the target group of the study, but outside the studied sample, were interviewed face to face. While providing the necessary explanations in each interview, their verbal opinions about the appropriateness, level of difficulty and comprehensibility of each item in the questionnaire were collected and the necessary modifications were applied based on the feedback of the target group.

Then face validity was done quantitatively. The questionnaire was given to the same 20 people to express their opinions about the importance of each of the questions and statements in a 5-point Likert scale from “not at all important” (score 1) to “very important” (score 5). Then, by calculating the result of importance coefficient in relative frequency, Impact score of each item was determined. Items with a score equal to or greater than 1.5 were retained in the questionnaire.

### Content validity

In order to attain content validity both qualitative and quantitative methods were used. For qualitative analysis 10 experts (in an expert panel) from the fields of health education (5 people), nutrition (2 people), statistics (1 person), and epidemiology (2 people) were consulted. They were asked to precisely analyze and express their opinions and give comments in regards to the instruments, correct syntax, correct grammar, appropriate scoring, time needed to design and make the needed instruments, consistency of the chosen guidelines, and the correct placement of chosen items. Experts’ comments were used to make the final intruments. For qualitative content validation, initially 10 experts were asked to evaluate each question with three choices (“necessary”, “useful but not necessary”, “not necessary”) and thus the content validity ratio (CVR) was calculated. Based on the Lawsche table, items with a CVR greater than 0.62 (*p* < 0.05) were kept [23].

$n_E$ : number of experts who considered the item necessary. 

$\frac N2$ : half of all the evaluations

$$CVR=\;\frac{n_E-{\displaystyle\frac N2}}{\displaystyle\frac N2}$$

Later, ten of the afore-mentioned experts were asked to give their opinions about three aspects of the questionnaire: inclusiveness, simplicity, and clarity on Likert scales. For instance, for the clarity criterion the following choices were used: “not clear” scored 1, “somewhat clear” scored 2, “clear” scored 3, and “completely clear” scored 4. Then, the content validity index (CVI) for each item was calculated by dividing the number of experts giving a score of 3 or 4 to the item by the number of all experts. Accordingly, only the items with a CVI higher than 0.79 were deemed as acceptable [24].

### Construct validity

In a cross-sectional study aimed to analyze the items and identify the main features of the questionnaire and also to satisfy its validity, classical item analysis and exploratory factor analysis were used. A multi-step cluster sampling method was also used. In order to expand the social and financial characteristics coverage of the target population, firstly Khorramabad city was divided into five postal regions including north, south, east, west, and center. Secondly, from each region a comprehensive Health Services Center was randomly included in the study. A list of all the diabetic patients was tabulated using the patient medical records of each health center. Random sampling, based on the needed target sample size and via random number table was used to complete the final list of patients in the study. In order to calculate the number of the necessary participants for the study, the number of items(60) was multiplied by 5 (5*6 = 300). A pilot study was conducted on 300 people for factor analysis of the questionnaire [25]. Due to the prevalence of the COVID-19 virus pandemic, the questionnaire was filled over the telephone. The acquired data were analyzed using IBM SPSS software (v. 26.0).

### Participants

Inclusion criteria include women and men aged 18 to 60 living in Khorramabad city, being diagnosed with diabetes at least 3 months prior to the study, having active medical records in the chosen heath centers, having complete knowledge and willingness to take part in the study, not being afflicted with advanced states of diabetes such as liver or kidney failure, being treated with oral blood glucose decreasing drugs, and not being diagnosed with known psychological disorders. The exclusion criteria included the patient’s unwillingness to continue cooperating in the study and incomplete completion of the questionnaires.

### Classical item analysis

To determine the internal stability of the instrument, the Corrected Item-Total Correlation method was used. In this method the correlation of each question with the complete instrument are measured and accordingly decisions are made to remove some unnecessary questions. For this purpose, the minimum criterion of this index was considered for the selection of items [26].

### Exploratory factor analysis

First, Kaiser-Meyer-olkin (KMO) and Bartlett sphericity tests were performed to evaluate the adequacy of the sample size and the correlation between the extracted factors. Then, to identify the main factors, exploratory factor analysis was used with varimax rotation with a cut-point of at least 0.3. And finally, the eigen value was used at least once. The Scree test was also used to confirm the identified factors [27].

### Reliability

In this study, Cronbach’s alpha coefficient was used to assess the consistency or internal consistency of the questionnaire to determine its reliability [28].

## Results

### Preliminary study results

The demographic characteristics of the subjects of this study are listed in Table 1.

**Table 1:** Frequency distribution of demographical characteristics of the subjects

Variable	**Status**	**Number**	**Percentage**
Sex	male	120	41.7
	female	168	58.3
nationality	lor	265	92
	Persian	13	4.5
	lak	10	3.5
Marital status	single	21	7.3
	married	263	91.3
	Divorced or widowed	4	1.4
education	illiterate	72	25
	Diploma or lower	110	38.2
	Academic education	106	36.8
occupation	employed	85	29.5
	housewife	151	52.4
	unemployed	52	18.1
Life Network Type	Living alone	13	4.5
	Living in a family	275	95.5
Family history of diabetes	yes	168	58.3
	no	120	41.7
Monthly income	low	109	37.8
	average	146	50.7
	good	28	9.7
	excellent	5	1.7
Body mass index	Low weight	8	2.8
	normal	96	33.3
	overweight	125	43.4
	fat	59	20.5

### Results of face validity

The total number of items in the initial questionnaire was 60 items. 6 items were removed due to having an impact score of less than 1.5. Thus, 54 items remained for the next stage.

### Results of quantitative content analysis

Based on the results of CVI calculation, out of 54 items examined, 4 items were removed due to not meeting the minimum score of 0.79 and a total of 50 items remained in the questionnaire. Consequently, as a result of calculating the CVR and according to the Lawsche table, 40 items remained and entered into the next stage of the psychometric process by removing 10 items.

### Results of classical item analysis

Out of the total of 40 items left from the previous stage, 5 items were removed due to not obtaining a minimum score of 0.3 from the CITC index and 1.96 from the skewness index, and finally 35 items remained (Table 2).

**Table 2:** Results of exploratory factor analysis of the subjects

**Items**	**Factor 1**	**Factor 2**	**Factor 3**	**Factor 4**	**Factor 5**	CITC Corrected Item- Total Correlation
	**Ability to read nutrition facts**	**Practical ability to group foods**	**Ability to calculate food rations**	**Ability to understand the impact of food on health**	**Ability to prepare food**	
If I want to know about the calories in a food package, I read its food label.	0.805					0.849
If I want to know about the health mark of a food package, I read its food label.	0.802					0.906
If I want to know about the validity of a food package company, I read its food label.	0.797					0.891
If I want to know about the food contents of the food package, I read its food label.	0.783					0.894
If I want to know about the amount of sugar, fat and salt in the food package, I read the label on it.	0.781					0.890
If I want to know about the expiration date of the food package, I will read the label on it.	0.581					0.658
What does the food pyramid represent?		0.737				0.494
What are the main food groups?		0.689				0.546
Which option is right for a healthy eating pattern in diabetics?		0.687				0.586
What does it mean to observe the principle of diversity in the food pyramid?		0.513				0.602
What does it mean to observe the principle of balance in the food pyramid?		0.496				0.501
What is the best choice of bread and cereals for diabetic patients?		0.468				0.482
What is the recommended daily allowance in the food pyramid for the vegetable group?			0.799			0.465
What is the recommended daily allowance in the food pyramid for the bread and cereal group?			0.780			0.702
What is the recommended daily allowance in the food pyramid for the fruit group?			0.771			0.406
What is the recommended daily allowance in the food pyramid for milk and milk products?			0.742			0.433
What is the recommended daily allowance in the food pyramid for the meat group and its substitutes?			0.722			0.429
Due to the high calorie content and lack of nutrients, which of the following options is not considered a food group?			0.582			0.302
What is the recommended daily intake of salt?			0.411			0.721
It is recommended to drink a few glasses of water a day:			0.392			0.419
Which balanced option is recommended as a snack in diabetics:				0.655		0.342
What are the best drinks for diabetics?				0.654		0.382
What is the advantage of using complex carbohydrates (such as whole grain breads and biscuits) over simple carbohydrates (such as sugar and sweets)?				0.649		0.352
What is the best healthy eating pattern for people with diabetes?				0.602		0.542
What is the most important principle of nutrition in diabetic patients?				0.502		0.652
What is the reason for the importance of consuming vegetables (tomatoes, lettuce, leeks, radishes) and legumes (peas, beans and lentils) in a healthy eating pattern in diabetic patients?				0.412		0.362
In the diabetic patient, which food group has consumption restrictions?				0.413		0.801
Why are ready-to-eat foods (such as pizza) and canned foods restricted in people with diabetes?				0.343		0.609
Which of the following diseases is associated with unhealthy nutrition?				0.334		0.521
In order to reduce calorie intake, which seasoning do you use to make salads and vegetables delicious?					0.845	0.601
In people with diabetes, what is tea best with?					0.779	0.770
How do you prepare meat and chicken?					0.461	0.641
How do you cook food to reduce the consumption of harmful fats?					0.341	0.640
**Eigenvalue**	2.916	2.650	1.912	1.587	1.280	
**Variance%**	8.836	8.030	5.795	4.810	3.878	
**Variance Cumulative %**	30.232	38.262	44.057	48.867	52.745	
**Factor Loading Range**	0.581–0.805	0.468–0.737	0.392–0.799	0.334–0.655	4.810–0.845	
**Correlation between** factor score and scale score						
** Cronbach’s Alpha**	0.951	0.785	0.741	0.640	0.610	
** Kaiser-Mayer-Olkin Measure of Sampling Adequacy**	0.836					
** Bartlet’s Test of Sphericity (Approx. Chi-Square)**	4212.142					
*** P* Value**	< 0.001					

### Results of exploratory factor analysis

The 35 remaining items from the previous stage were incorporated in this stage. Firstly, to ensure the adequacy of the sample size, the Kaiser-Meyer-olkin (KMO) test was performed. The Kaiser-Meyer-olkin criterion (KMO) was 0.836, indicating that the data are suitable for exploratory factor analysis. Bartlett sphericity test was also significant (*P* < 0.001: 4212.142) indicating there was sufficient correlation among the variables. As a result of factor analysis with an eigenvalue of at least one, 7 factors were identified, but some of these factors were weak and had 1–2 items. Therefore, factor analysis with an eigen value of at least 1.2 was repeated, which represented 35 items in the form of five factors. The factor load of the items was in the range of 0.805 − 0.334. The total variance of the 5-factor model was 52.745%. And the variance of each factor is shown in the table (Table 2). According to the concepts resulting from the items loaded on each factor, as well as by reviewing related texts and literature, the identified factors were named in the following order, the first factor was “the ability to search and read food facts”,which included 6 items. This factor was the strongest factor in food literacy in diabetic patients. This factor deals with the ability to look for information on food labels (for example, if I want to know about the expiration date of a food package, I read the label on it). The questions had multiple options: “always,” “most of the time,” “sometimes”, and “rarely” which were scored 1, 2, 3, and 4, respectively. The second factor was the “ability to group foods efficiently”, which included 6 items. This factor showed a person’s ability to identify the main food groups and the food pyramid. The third factor was the “ability to calculate food serve sizes ,” which consisted of 8 items, this is the most important component in diet management. This factor revolves around a person’s ability to calculate the recommended daily allowance in the food pyramid for different food groups (for example, what is the recommended daily allowance in the food pyramid for the fruit group?). The fourth factor was the “ability to understand the effect of food on health,” which consisted of 9 items and measured a person‘s ability of understanding or knowledge of dietary factors that can improve or prevent optimal health in people with diabetes (for example, the ability to identify the impact of foods that are high in sugar and fat and include the health benefits of fiber foods such as vegetables and fruits) the fifth factor, “healthy ability to prepare food in a healthy manner” included 4 items. This factor is a person’s ability to prepare and cook food in a way that has fewer calories and that is healthier (Table 2, Additional file).

The second, third, fourth, and fifth factors were scored in such a way that the questions consisted of 4 options, one of which was a correct option and the other options were incorrect. The correct option was assigned a score of one and the other options were assigned a score of zero.

The reliability of the instrument was calculated by factor analysis using Cronbach’s alpha for the whole questionnaire and also for each factor. The reliability coefficient for the whole instrument was calculated to be 0.83 and between 0.610 and 0.951 for the five factors (Table 2, Additional file).

## Discussion

The aim of this study was to develop and evaluate the validity and reliability of the Food Literacy Questionnaire in people with diabetes. The questionnaire consisted of 33 items in 5 dimensions including the ability to read food labels and facts, the ability to operationally group foods, the ability to calculate food serve sizes, the ability to understand the impact of food on health, and the ability to prepare healthy foods. The items of this questionnaire had an acceptable factor load in the range of 0.805 − 0.334, which explained 52.745% of its variation. Reliability was calculated for 5 factors between 0.951 − 0.610. For the questionnaire at hand the item impact score, CVR, and CVI of all items were respectively greater than 1.5, 0.62, and 0.79, confirming its acceptable face and content validities. In addition, classical item analysis used to assess construct validity showed that all items had a corrected item-total correlation coefficient greater than 0.3. In other words, the scales had acceptable internal consistency and construct validity. Moreover, the study findings also revealed that all items had an acceptable factor leading value greater than 0.3. This finding confirms that all items were important and the scale had acceptable construct validity [29–32].

Previous studies have shown that health literacy includes dimensions such as functional, interactive, and critical health literacy. Functional health literacy means understanding and using information. Interactive health literacy means having the ability to communicate and interact and find information. And critical health literacy means critical evaluation of information [13, 23, 33]. The factors explored in the present study correspond to the mentioned dimensions. The first factor was “the ability to read nutritional facts”. The ability to read food facts actually means the ability to find nutritional information about foodstuffs, usually by reading labels on food packages. According to a previous study [34], this factor indicates interactive food literacy, for example a person tries to find information about the food he/she consumes. This dimension was called the production dimension in a previous study, which examined food label items such as food components and ingredients [25]. Today, in many countries of the world, the labeling of foods to indicate nutritional information is considered mandatory. Raising nutritional awareness and knowledge about the role of diet in the control of chronic diseases such as cardiovascular disease, obesity, diabetes, and hypertension, has made people pay more attention to food selection and information collection about the nutritional values of various foodstuffs. Studying the available information on food packages, such as production and expiration dates and nutritional information labels containing information on the caloric content and some nutrients can also be effective in choosing healthy and safe foods and thus changing the nutritional behavior of consumers [35, 36]. However, based on the findings of various studies, consumers mainly pay attention to issues such as convenience, price differences, etc., which are much more important and significant for them than paying attention to food labels and nutritional information also available on food labels [37]. Attention to nutritional information on food labels was reported to be 78% [38] in Americans and 66% [39] in Koreans. A study carried out on Iranians showed that only 4.6% read the labels for the purpose of obtaining nutritional information, and 66.7% only read the date of production and expiration of the product [40]. This study showed that being written in fine print, disbelief in the importance of nutritional information, lack of interest, lack of clarity, lack of literacy, or lack of time were the reasons why consumers did not pay attention to food labels. Knowledge of the information on food packages can influence people with diabetes to make purchasing decisions and, as a result, change their eating habits toward a healthy and desirable eating pattern.

The second, third, and fifth dimensions, under the heading “The ability to operationally group foods”, “the ability to calculate food serve sizes”, and “the ability to prepare healthy food “refer to the understanding and use of information that, according to previous studies [1, 20–22] fall into the functional literacy category. The ability to operationally group foods measures a person’s food literacy on the type of food groups and the principles of diversity and balance in the diet. The level of nutritional awareness is significantly related to nutritional behaviors and nutritional awareness is one of the factors that in addition to the individual, also affects the eating habits of the family and those around him. Studies carried out by Karimi et al. and Moynihan et al. showed that nutritional attitudes and beliefs are important factors in predicting nutritional behavior and performance [41, 42]. The results of Videga et al.’s study on pregnant women in the United States also show better and more appropriate intake of energy, folate, vitamin B6, iron, zinc, and calcium, as well as the number of daily meals consumed from the group of vegetables, bread, and cereals in pregnant women with higher food literacy [43]. Healthy eating is achieved by observing the two principles of balance and variety in the daily diet plan. Balance means consuming sufficient amounts of nutrients needed to maintain physical health, and diversity means consuming different types of foods that are classified in the 5 main food groups.

The third factor, called the “ability to calculate food serve sizes”, measures people’s literacy about the food pyramid. This dimension were called “intake “in a previous study, which measures things like having meals from all relatively balanced food groups [25]. The Healthy Food Pyramid is a simple guide to the types and proportions of nutrients that should be consumed on a daily basis by people to enjoy a healthy life. The food pyramid is the best guide to adjust eating habits. Nowadays, nutritionists try to present nutritional knowledge to all people in the form of food guides and use the food pyramid as a scientific and effective tool to teach proper nutrition. The food pyramid is designed based on the three principles of balance, diversity and the embodiment of proportions in the selection of food groups. The 5 food groups of the pyramid include bread and cereals, vegetables and fruits, milk and dairy products, and meat and its substitutes. If these food groups are consumed in certain amounts, they will make up a healthy diet. This instrument has two main uses. It provides a good model for people to receive daily food in order to provide energy and nutrients. Limits the use of substances that increase the risk of chronic and non-communicable diseases such as diabetes.

The fourth factor was “the ability to understand the impact of food on health”. This dimension is about maintaining a healthy diet in diabetic people. The need to be able to evaluate and analyze nutritional information is more important in diabetic patients compared to other people. Accordingly, it is categorized as critical food literacy which means critical evaluation of information. This dimension was called “selection” in a previous study. It measures the ability to understand information about diet appropriate to one’s situation [25]. The results of a previous study show that 65% of people are unaware of the dangers of consuming too much fat and the exacerbating effect it has on diseases. This lack of knowledge about the type of food that should be consumed is obvious, because some people either avoid eating healthy foods or have difficulty in choosing it [44]. Following a diet is an important part of the treatment plan for patients with type 2 diabetes, in which it is recommended to reduce the intake of simple sugars, saturated fats, cholesterol, increase the intake of fruits and vegetables and dietary fiber. In many cases, these patients do not agree with these recommendations due to lack of knowledge.

The fifth factor is the ability of diabetic patients to prepare low-calorie healthy foods. This dimension was entitled preparation and cooking in a previous study, which examines the observance of health in the preparation and cooking of food, such as healthy cooking recipes, the use of healthy condiments, and the preparation and storage of foods in a healthy manner [25].According to this study, this dimension falls into the functional category of health literacy. A previous study showed that there is a statistically meaningful relationship between nutritional skills, diet decision making, eating problems and dietary barriers with steps to prepare to change one’s diet [45]. Food preparation skills can enable diabetic patients to make good food choices and improve their metabolic status and quality of life.

The limitation of the present study was that it was conducted during the outbreak of the Covid-19 disease. For this reason, the transportation for people with diabetes was limited and the questionnaire was completed through a telephone interview, which reduced their participation in this study.

## Conclusion

The item impact score, CVR and CVI of all 33 items on the scale were higher than 1.5, 0.62 and 0.79, respectively, which confirms its formal validity and acceptable content. The results of this study showed that the exploratory dimensions in the present study are consistent with the dimensions of health literacy including functional, interactive, and critical food literacy. The ability to read nutritional facts indicates interactive food literacy: that the person is trying to find information about the food they are eating. The dimensions of “ability to group food efficiently”, “ability to calculate serve sizes”, and “ability to prepare healthy foods” refer to the understanding and use of information that fall into the category of functional literacy. The “ability to understand the effect of food on health” dimension is about healthy eating in diabetic patients who need to evaluate and analyze nutritional information in this group of people. Therefore, it is classified in the critical food literacy category which means critical evaluation of information. Health professionals can use this scale to assess and promote food literacy in people with diabetes.

## Supplementary Information

**Additional file 1.** 

## Data Availability

The datasets used and/or analyzed during the current study are available from the corresponding author on reasonable request.

## Abbreviations

CVR: Content validity ratio
CVI: Content validity index
KMO: Kaiser-Meyer-Olkin test
CITC: Classical Item Analysis
