# Supplementary material for: Development and psychometric analysis of a new tool to assess food literacy in diabetic patients
Source: BMC Nutr. 2022 Nov 16;8:134. doi: 10.1186/s40795-022-00626-4 (PMC9666971; doi:10.1186/s40795-022-00626-4)
Supplement: Supplementary file 1 — Additional file 1. [file 40795_2022_626_MOESM1_ESM.docx]

**Description of data**

**Factor 1: Ability to read nutrition facts**

1. If I want to know about the calories in a food package, I read its food label.

a. rarely

b.some times

c.most of the time

d.Always

1. If I want to know about the health mark of a food package, I read its food label.

a.rarely

b.sometimes

c.most of the time

d.Always

1. If I want to know about the validity of a food package company, I read its food label.

a.rarely

b.sometimes

c.most of the time

d.Always

1. If I want to know about the food contents of the food package, I read its food label.

a.rarely

b.sometimes

c.most of the time

d.Always

1. If I want to know about the amount of sugar, fat and salt in the food package, I read the label on it.

a.rarely

b.sometimes

c.most of the time

d.Always

1. If I want to know about the expiration date of the food package, I will read the label on it.

a.rarely

b.sometimes

c.most of the time

d.always

**Factor 2: Practical ability to group foods**

1. What does the food pyramid represent?

a. A certain amount of fruit consumption

b. A certain amount of dairy consumption

c.. A certain amount of fat consumption

d. Definite intake of all food groups

2. What are the main food groups?

a. Bread and cereals group

b.Fruits and vegetables

c.Meat, legumes, eggs and nuts

d. All items plus milk and dairy group

3. Which option is right for a healthy eating pattern in diabetics?

a.Variety in the diet

b.Balance in the diet plan

c.Diversity and balance in the diet

d.I do not know

4. What does it mean to observe the principle of diversity in the food pyramid?

a. Daily consumption of different types of food

b.Consume a variety of vegetables

c.Consume a variety of fruits

d.I do not know

5. What does it mean to observe the principle of balance in the food pyramid?

a.Consume sufficient amounts of required ingredients

b.Adequate consumption of meat and dairy products

c.Excessive consumption of vegetables and fruits

d.I do not know

6. What is the best choice of bread and cereals for diabetic patients?

a.Sangak bread and other whole grain breads

White rice and baguette bread

White rice and barbarian bread

Pasta and lavash bread

**Factor 3: Ability to calculate food rations**

1. What is the recommended daily allowance in the food pyramid for the vegetable group?

5-3 Daily share .a

4-2 daily share.b

Consume freely.c

I do not know.d

2. What is the recommended daily allowance in the food pyramid for the bread and cereal group?

a. Daily consumption of 11-6 shares

b.Daily consumption of 8-6 shares

c.Consume freely

d.Less than other food groups

3. What is the recommended daily allowance in the food pyramid for the fruit group?

4-2 daily share.a

5-3 daily share.b

Consume freely.c

I do not know.d

4. What is the recommended daily allowance in the food pyramid for milk and milk products?

a.Fertile and lactating women 4-3 daily shares, other people 2-3 daily shares

b.In all people 2-3 shares daily

c.In all people 4-3 daily shares

d.I do not know

5. What is the recommended daily allowance in the food pyramid for the meat group and its substitutes?

3-2 Daily share.a

Less than 2 shares daily.b

Consume freely.c

I do not know.d

6. Due to the high calorie content and lack of nutrients, which of the following options is not considered a food group?

a.Meat

b.Animal fats, sweets, liquid and solid oils

c.Fruits and vegetables

d.dairy

7. What is the recommended daily intake of salt?

a.Maximum 5 grams daily (about the size of a tablespoon of jam)

b.Maximum 10 grams daily (about two tablespoons of jam)

c.Maximum 15 grams per day (about three tablespoons of jam)

d.I do not know

8. It is recommended to drink a few glasses of water a day:

6-8 glasses daily.a

As long as my thirst is quenched.b

4 glasses.c

I do not know.d

**Factor 4: Ability to understand the impact of food on health**

1. Which balanced option is recommended as a snack in diabetics:

a. Whole meal biscuits and nuts

B. Consumption of fruits and vegetables

C. milk

D. all items

2. What are the best drinks for diabetics?

a. Low salt water and butter without gas

b.Packaged juices

c.Carbonated drinks

d.I do not know

3. What is the advantage of using complex carbohydrates (such as whole grain breads and biscuits) over simple carbohydrates (such as sugar and sweets)?

a. Complex carbohydrates raise blood sugar faster

B. Complex carbohydrates raise blood sugar more slowly

C. It is easier to digest and absorb complex carbohydrates

D. It is more difficult to digest and absorb simple carbohydrates

4. What is the best healthy eating pattern for people with diabetes?

a. Eat three main meals at the appointed time

B. Eat three main meals at the appointed time and one snack

c. Eat two main meals at the appointed time and two snacks

D. Eat three main meals at the appointed time and three snacks

5. What is the most important principle of nutrition in diabetic patients?

a. Complete elimination of sugars and sweets and cereals

B. Use natural sugars (dates and raisins) as desired

c. Arbitrary consumption of fruits

D. Proportional and balanced use of the main food groups based on the food pyramid

6. What is the reason for the importance of consuming vegetables (tomatoes, lettuce, leeks, radishes) and legumes (peas, beans and lentils) in a healthy eating pattern in diabetic patients?

a. The fiber in them helps control blood sugar better

B. The fiber in them makes you feel full

C. Option 1 and 2

D. I do not know

7. In the diabetic patient, which food group has consumption restrictions?

a. Meat group and its substitutes

B. Animal fats and solid oils

C. Simple sugars (such as sugar) and foods containing such sugars

D. Option 2 and 3

8. Why are ready-to-eat foods (such as pizza) and canned foods restricted in people with diabetes?

a. They contain a lot of salt

b.They contain a lot of salt and fat

c.They contain a lot of sugar, salt and fat

d.I do not know

9. Which of the following diseases is associated with unhealthy nutrition?

a. Incidence of gastrointestinal cancers

b.Cardiovascular diseases and diabetes

c.Osteoporosis

d.all items

**Factor 5: Ability to prepare food**

1. In order to reduce calorie intake, which seasoning do you use to make salads and vegetables delicious?

a.I do not know

b.mayonnaise sauce

c.Lemon juice and orange juice

d.Lemon juice and orange juice and some oil

2. In people with diabetes, what is tea best with?

a.I do not know

b.Balanced consumption of sugar

c.Balanced consumption of dates

d.Balanced consumption of dried fruits such as raisins, berries and figs

3. How do you prepare meat and chicken?

a.I remove the fat from the meat and chicken skin

b.I do not see the need to separate the fat from the meat and chicken skin

c.I just peel the chicken skin

d.I do not know

4. How do you cook food to reduce the consumption of harmful fats?

a.Steaming foods

b.Boil food

c.frying

d.Options 1 and 2
